# Advanced Characterization of Adhesive Joints and Adhesives

**DOI:** 10.3390/ma15207347

**Published:** 2022-10-20

**Authors:** Raul Duarte Salgueiral Gomes Campilho, Kouider Madani, Chander Prakash

**Affiliations:** 1 ISEP—School of Engineering, Polytechnic of Porto, R. Dr. António Bernardino de Almeida, 431, 4200-072 Porto, Portugal; 2 INEGI—Institute of Science and Innovation in Mechanical and Industrial Engineering, Pólo FEUP, Rua Dr. Roberto Frias, 400, 4200-465 Porto, Portugal; 3 Department of Mechanical Engineering, University of Sidi Bel Abbes, BP 89 Cité Ben M’hidi, Sidi Bel Abbes 22000, Algeria; 4 School of Mechanical Engineering, Lovely Professional University, Phagwara, Punjab 144411, India

Structural adhesives have shown significant improvements in their behavior over the past few decades. This has enabled their application to become a reality in many sectors of activity, including the aeronautics and the automotive industry [1]. This evolution has been strongly supported by an intense investigation into adhesive joints and their behavior. Despite this intense research, there is still much to be explored regarding this matter, which translates into a continuous investigation of the failure modes of these types of joints, the characterization of new adhesives, the design of new joint geometries, and the use of hybrid joints, with a view to eliminating or reducing the less positive aspects presented by these joints, taking advantage of the best characteristics of each type of joint. Numerical methods have played an extremely important role in the prediction of the joints’ behavior, helping to find the best solutions to the typical problems presented by these kinds of joints [2]. Strength prediction techniques can be mainly divided into static and dynamic, with the former being subjected to a wider research effort from the academic community. Nonetheless, recently, significant efforts have been made to address complex dynamic loadings, such as fatigue and impact.

Static techniques can be either analytical or numerical. Due to the simplifying assumptions, analytical techniques are usually restricted to initial evaluations or basic joint geometries, which are seldom used in real applications. Numerical techniques have a more widespread use and enable the analysis of complex joints. The relevant static modeling alternatives are described next.

- Different analytical techniques were proposed in the literature in the last few decades for strength prediction of bonded joints. These techniques estimate stresses/strains in the adhesive layer, and failure is then assessed by comparison with the adhesive properties. The review of da Silva et al. [3,4] addresses analytical techniques up to 2009, and performs a detailed comparison and accuracy assessment. The review of Ramalho et al. [5] extends the latest findings up to 2020. Recent approaches on this topic include references [6,7].
- Continuum mechanics techniques are typically coupled with finite element method (FEM) analyses to extract stresses in the adhesive layer and predict the joint strength [8]. A few examples of this technique are proposed in recent works [9,10]. The main limitations of this approach appear in the form of stress singularities at the sharp corners, whose stresses do not converge with the reduction in the elements’ size. Recently, new failure criteria were proposed to be used with this approach, such as the critical longitudinal strain (CLS) criterion [11] or the critical normal strain (CNS) criterion [12], with accurate results for specific joints.
- Conventional fracture mechanics evaluates discontinuities in materials, which is not possible to accomplish with continuum mechanics. In bonded joints, these geometrical perturbations appear as corners at the overlap ends or even defects. Stress intensity factors (SIF) and strain energy release rates (SERR) constitute the two main approaches [13]. Cameselle-Molares et al. [14] applied the virtual crack closure technique (VCCT) for strength evaluation of double-lap bonded joints in composite structures. Although the results were good and agreed with the experimental tests, the model is only valid for brittle adhesives, since the model does not include plastic deformations. The recent finite fracture mechanics (FFM) criterion [15,16] estimates crack onset by satisfying an energetic and a stress criterion. This criterion only applies to brittle adhesives.
- Damage mechanics simulates adhesive degradation by stiffness reduction in the finite elements, up to reaching failure or complete loss of stiffness. By this approach, it is possible to reproduce arbitrary crack paths in the materials. Stapleton et al. [17] developed a FEM/damage mechanics formulation with adaptative shape functions and mesh. This formulation took advantage of a reduced number of elements, thus being more efficient than cohesive zone modeling (CZM), while providing equally accurate results.
- CZM is currently the most powerful technique for static fracture modeling of adhesive joints [18,19]. The cohesive laws take advantage of mixed-mode stress criteria to infer damage initiation and fracture criteria for crack propagation. CZM excels in mesh independent strength predictions, since damage growth is triggered by an energetic criterion averaged over an area, instead of stress-based concepts [20]. An original approach was recently proposed by Geleta et al. [21], who discretized the adhesive layer of inclined adhesive joints with continuum elements and inserted cohesive elements between all of the layers. The numerical results were in good correspondence with the test results. In recent years, complex CZM shapes were proposed to increase accuracy over the traditional CZM shapes, such as the Park–Paulino–Roesler (PPR) law [22], which includes a parameter to vary the softening curvature, thus enabling the simulation of varying adhesive plasticity.
- The extended finite element method (XFEM) was developed in 1999 [23] and consists of an FEM basis and application of enriched displacement fields near the crack. The XFEM was initially tested in generic fracture mechanics problems, and its application to adhesive joints is relatively new. Overall, the XFEM advantage over CZM is related to mesh independent crack propagation [24]. Machado et al. [25] studied stepped-lap joints with different overlap lengths by the XFEM. Accurate maximum load predictions were found by correctly choosing the damage initiation criteria, with errors lower than 10%. Mubashar et al. [24] proposed a mixed XFEM-CZM model to analyze single-lap joints with adhesive fillets at the overlap ends, by considering a triangular cohesive law for the adhesive/adherend interfaces, and XFEM-enriched elements for the bondline and fillet adhesive portions. Damage initiation in the XFEM elements was triggered by the maximum principal stress criterion, leading to precise maximum load and crack path predictions.
- Meshless methods excel in fracture modeling by not needing to remesh, but these are not explored in the context of bonded joints. In meshless methods, the nodes connect by using influence domains instead of FEM elements. Tsai et al. [26] applied the symmetric smoothed particle hydrodynamics meshless method with CZM to fracture prediction in double-cantilever beam (DCB) specimens, under different loading modes. The results for mode I and mixed-mode loads were accurate.

Regarding future trends, it is expected that CZM will benefit from improved calibration tools that facilitate industrial applications. XFEM has limitations, such as crack paths, which could be improved for the purpose of bonded joints. Meshless methods for adhesive joints are incipient, but the inclusion of fracture criteria to simulate damage growth would enable these to complete with the established techniques.

Dynamic loadings are the focus of a large amount of published works due to increasing academic and industrial interest, and there are three main research lines, which are as follows: fatigue, variable strain rate and impact, and modal analysis [27].

- For fatigue analysis, continuum mechanics is the simplest approach. Its use can, thus, be considered in preliminary estimations, although the precision is not the best. This approach consists of estimating the number of cycles to failure (life) using stresses/strains in the adhesive layer. The work of Esmaeili et al. [28] compared different continuum models in bolted and hybrid (bonded/bolted) double-lap joints. Between models, the stress-based Crossland model provided the closest fatigue life predictions to reality. Fracture mechanics techniques have wide applications for fatigue, since they account for crack growth. Fatigue crack propagation can be implemented with SIF or SERR, with an emphasis on the latter technique. Wu et al. [29] recently applied the J-integral for SERR prediction of hybrid (bonded/riveted) joints, showing a SERR reduction when the crack in the adhesive approaches the rivet. The VCCT was used by Pascoe et al. [30] to assess the adhesive thickness effect in pure-mode I fatigue tests using the DCB test. The models revealed an increase in the plastic zone with the adhesive thickness, leading to higher dissipation of plastic energy. CZM is the most addressed technique nowadays for fatigue life assessment. Under this scope, continuum mechanics or strength of materials is considered for damage initiation, and crack growth is modeled by fracture concepts, such as the Paris laws. Ebadi-Rajoli et al. [31] modeled fatigue crack propagation in pure and mixed-mode fracture specimens with triangular CZM, and managed to reproduce the experimental Paris’ law curve.
- Variable strain rate and impact loadings can be addressed with continuum mechanics. However, it is known that, under impact loading, stress propagation waves arise [32]. Nonetheless, failure normally takes place at the edges of the adhesive layer. Quaglini et al. [33] numerically analyzed stresses in glass plates with point fixings and the following two different joining types: bolting and adhesive bonding. It was shown that bolting gives rise to significant peak stresses in the glass, in contrast to what occurs with adhesive bonding. Damage mechanics is another modeling option to simulate material degradation and failure along arbitrary paths in the structure. Gollins et al. [34,35] addressed a near mode I joint under impact by damage mechanics, both experimentally and numerically. The proposed damage model precisely predicted the impact strength for two adhesives and different impact speeds. CZM is also recognized as a predictive technique for high strain rates and impact. The analysis procedure for impact is identical to the static case, although the strain-rate dependency of CZM parameters must be included in the models. As a result, the adhesive properties should be tested under the same strain rate of the joint tests [36]. Lißner et al. [37,38] proposed trapezoidal CZM that included the effects of loading rate and adhesive thickness. The experimental tests enabled the characterization of the CZM laws and revealed an increase in toughness with the adhesive thickness for tensile, shear and mixed-mode loadings. Peak stresses increased with the testing rate, while the toughness decreased. Valente et al. [39] compared the behavior of three adhesives in single-lap joints under impact, considering steel adherends and triangular CZM. The model was positively validated with experimental data, and the results showed a significant adhesive-type effect, with mechanically stronger adhesives leading to improved performance.
- Modal analysis deals with natural vibration frequencies and respective vibration modes, which is pertinent, since structures should work outside those frequencies. Since adhesives have damping properties, these can be used to improve structural functionality for specific operation conditions. Almitani and Othman [40] implemented an analytical vibration model for both single- and double-lap joints, and carried out FEM validation. Challita [41] proposed and validated by the FEM an analytical model for single-lap joints with voids in the adhesive. The natural frequencies agreed with the FEM results.

Analysis of dynamic loading cases showed that natural frequencies and damping are not sufficiently addressed in the literature and could benefit from more attention. Numerical works usually rely on the FEM, in contrast to methods such as the XFEM or meshless methods. Thus, future works dedicated to the dynamic behavior of bonded joints can be tested with alternative methods to verify their ability for these conditions. Meshless methods, for instance, could be particularly useful to induce crack growth in the models, since these do not require remeshing.

## References

[1] Adams R.D. (2005). Adhesive Bonding: Science, Technology and Applications.

[2] Campilho R.D. (2017). Strength Prediction of Adhesively-Bonded Joints.

[3] da Silva L.F.M., das Neves P.J.C., Adams R.D., Spelt J.K. (2009). Analytical Models of Adhesively Bonded Joints—Part I: Literature Survey. Int. J. Adhes. Adhes..

[4] da Silva L.F.M., das Neves P.J.C., Adams R.D., Wang A., Spelt J.K. (2009). Analytical Models of Adhesively Bonded Joints—Part II: Comparative Study. Int. J. Adhes. Adhes..

[5] Ramalho L.D.C., Campilho R.D.S.G., Belinha J., da Silva L.F.M. (2020). Static strength prediction of adhesive joints: A review. Int. J. Adhes. Adhes..

[6] Weißgraeber P., Stein N., Becker W. (2014). A general sandwich-type model for adhesive joints with composite adherends. Int. J. Adhes. Adhes..

[7] de Sousa C.C.R.G., Campilho R.D.S.G., Marques E.A.S., Costa M., da Silva L.F.M. (2017). Overview of different strength prediction techniques for single-lap bonded joints. J. Mater. Des. Appl. Part L.

[8] Spaggiari A., Castagnetti D., Dragoni E. (2012). Experimental Tests on Tubular Bonded Butt Specimens: Effect of Relief Grooves on Tensile Strength of the Adhesive. J. Adhes..

[9] Gültekin K., Akpinar S., Özel A., Öner G.A. (2017). Effects of unbalance on the adhesively bonded composites-aluminium joints. J. Adhes..

[10] Liu Y., Lemanski S., Zhang X. (2018). Parametric study of size, curvature and free edge effects on the predicted strength of bonded composite joints. Compos. Struct..

[11] Ayatollahi M.R., Akhavan-Safar A. (2015). Failure load prediction of single lap adhesive joints based on a new linear elastic criterion. Theor. Appl. Fract. Mech..

[12] Razavi S.M.J., Ayatollahi M.R., Majidi H.R., Berto F. (2018). A strain-based criterion for failure load prediction of steel/CFRP double strap joints. Compos. Struct..

[13] da Silva L.F.M., Campilho R.D.S.G. (2012). Advances in Numerical Modelling of Adhesive Joints.

[14] Cameselle-Molares A., Sarfaraz R., Shahverdi M., Keller T., Vassilopoulos A.P. (2017). Fracture mechanics-based progressive damage modelling of adhesively bonded fibre-reinforced polymer joints. Fatigue Fract. Eng. Mater. Struct..

[15] Leguillon D. (2002). Strength or toughness? A criterion for crack onset at a notch. Eur. J. Mech. A/Solids.

[16] Le Pavic J., Stamoulis G., Bonnemains T., da Silva D., Thévenet D. (2019). Fast failure prediction of adhesively bonded structures using a coupled stress-energetic failure criterion. Fatigue Fract. Eng. Mater. Struct..

[17] Stapleton S.E., Pineda E.J., Gries T., Waas A.M. (2014). Adaptive shape functions and internal mesh adaptation for modeling progressive failure in adhesively bonded joints. Int. J. Solids Struct..

[18] Sugiman S., Ahmad H. (2017). Comparison of cohesive zone and continuum damage approach in predicting the static failure of adhesively bonded single lap joints. J. Adhes. Sci. Technol..

[19] Ferreira C.L., Campilho R., Moreira R.D.F. (2020). Experimental and numerical analysis of dual-adhesive stepped-lap aluminum joints. Proc. Inst. Mech. Eng. Part E J. Process Mech. Eng..

[20] Rocha R.J.B., Campilho R.D.S.G. (2018). Evaluation of different modelling conditions in the cohesive zone analysis of single-lap bonded joints. J. Adhes..

[21] Geleta T.N., Woo K., Cairns D.S., Samborsky D. (2018). Failure behavior of inclined thick adhesive joints with manufacturing defect. J. Mech. Sci. Technol..

[22] Park K., Paulino G.H., Roesler J.R. (2009). A unified potential-based cohesive model of mixed-mode fracture. J. Mech. Phys. Solids.

[23] Moës N., Dolbow J., Belytschko T. (1999). A finite element method for crack growth without remeshing. Int. J. Numer. Methods Eng..

[24] Mubashar A., Ashcroft I.A., Crocombe A.D. (2014). Modelling damage and failure in adhesive joints using a combined XFEM-cohesive element methodology. J. Adhes..

[25] Machado R.M.D., Campilho R.D.S.G., Rocha R.J.B. (2019). Extended finite element modelling of aluminium stepped-adhesive joints. J. Adhes..

[26] Tsai C.L., Guan Y.L., Ohanehi D.C., Dillard J.G., Dillard D.A., Batra R.C. (2014). Analysis of cohesive failure in adhesively bonded joints with the SSPH meshless method. Int. J. Adhes. Adhes..

[27] Ramalho L.D.C., Sánchez-Arce I.J., Gonçalves D.C., Belinha J., Campilho R.D.S.G. (2022). Numerical analysis of the dynamic behaviour of adhesive joints: A review. Int. J. Adhes. Adhes..

[28] Esmaeili F., Zehsaz M., Chakherlou T.N., Barzegar S. (2015). Fatigue life estimation of double lap simple bolted and hybrid (bolted/bonded) joints using several multiaxial fatigue criteria. Mater. Des..

[29] Wu G., Li D., Lai W.-J., Shi Y., Kang H., Peng Y., Su X. (2021). Fatigue behaviors and mechanism-based life evaluation on SPR-bonded aluminum joint. Int. J. Fatigue.

[30] Pascoe J.A., Zavatta N., Troiani E., Alderliesten R.C. (2020). The effect of bond-line thickness on fatigue crack growth rate in adhesively bonded joints. Eng. Fract. Mech..

[31] Ebadi-Rajoli J., Akhavan-Safar A., Hosseini-Toudeshky H., da Silva L.F.M. (2020). Progressive damage modeling of composite materials subjected to mixed mode cyclic loading using cohesive zone model. Mech. Mater..

[32] Yildiz S., Andreopoulos Y., Delale F., Smail A. (2020). Adhesively bonded joints under quasi-static and shock-wave loadings. Int. J. Impact Eng..

[33] Quaglini V., Cattaneo S., Biolzi L. (2020). Numerical assessment of laminated cantilevered glass plates with point fixings. Glass Struct. Eng..

[34] Gollins K., Elvin N., Delale F. (2020). Characterization of adhesive joints under high-speed normal impact: Part I—Experimental studies. Int. J. Adhes. Adhes..

[35] Gollins K., Elvin N., Delale F. (2020). Characterization of adhesive joints under high-speed normal impact: Part II—Numerical studies. Int. J. Adhes. Adhes..

[36] Li Y., Yang Y., Li J., Wang B., Liao Y. (2020). Experimental-numerical analysis of failure of adhesively bonded lap joints under transverse impact and different temperatures. Int. J. Impact Eng..

[37] Lißner M., Alabort E., Cui H., Pellegrino A., Petrinic N. (2018). On the rate dependent behaviour of epoxy adhesive joints: Experimental characterisation and modelling of mode I failure. Compos. Struct..

[38] Lißner M., Alabort E., Cui H., Rito R., Blackman B.R.K., Petrinic N. (2019). Experimental characterisation and numerical modelling of the influence of bondline thickness, loading rate, and deformation mode on the response of ductile adhesive interfaces. J. Mech. Phys. Solids.

[39] Valente J.P.A., Campilho R.D.S.G., Marques E.A.S., Machado J.J.M., da Silva L.F.M. (2019). Adhesive joint analysis under tensile impact loads by cohesive zone modelling. Compos. Struct..

[40] Almitani K.H., Othman R. (2016). Analytical solution of the harmonic response of visco-elastic adhesively bonded single-lap and double-lap joints. Int. J. Adhes. Adhes..

[41] Challita G. (2018). Analytical study of the dynamic behavior of a voided adhesively bonded lap joint under axial harmonic load. Int. J. Solids Struct..

